# Shared decision making and antibiotic benefit-harm conversations: an observational study of consultations between general practitioners and patients with acute respiratory infections

**DOI:** 10.1186/s12875-018-0854-y

**Published:** 2018-10-06

**Authors:** Mina Bakhit, Chris Del Mar, Elizabeth Gibson, Tammy Hoffmann

**Affiliations:** Centre for Research in Evidence-Based Practice (CREBP), Faculty of Health Sciences and Medicine, Bond University, Robina, 4229 Australia

**Keywords:** Decision making, General practice, Respiratory tract infections, Decision support techniques, Physician-patient relations

## Abstract

**Background:**

Little research has examined whether shared decision making (SDM) occurs in consultations for acute respiratory infections (ARIs), including what, and how, antibiotic benefits and harms are discussed. We aimed to analyse the extent and nature of SDM in consultations between GPs and patients with ARIs, and explore communication with and without the use of patient decision aids.

**Methods:**

This was an observational study in Australian general practices, nested within a cluster randomised trial of decision aids (for acute otitis media [AOM], sore throat, acute bronchitis) designed for general practitioners (GPs) to use with patients, compared with usual care (no decision aids). Audio-recordings of consultations of a convenience sample of consenting patients seeing a GP for an ARI were independently analysed by two raters using the OPTION-12 (observing patient involvement in decision making) scale (maximum score of 100) and 5 items (about communicating evidence) from the Assessing Communication about Evidence and Patient Preferences (ACEPP) tool (maximum score of 5). Patients also self-completed a questionnaire post-consultation that contained items from CollaboRATE-5 (perceptions of involvement in the decision-making process), a decisional conflict scale, and a decision self-efficacy scale. Descriptive statistics were calculated for each measure.

**Results:**

Thirty-six consultations, involving 13 GPs, were recorded (20 for bronchitis, 10 sore throat, 6 AOM). The mean (SD) total OPTION-12 score was 29.4 (12.5; range 4–54), with item 12 (need to review decision) the highest (mean = 3) and item 10 (eliciting patients’ preferred level of decision-making involvement) the lowest (mean = 0.1). The mean (SD) total ACEPP score was 2 (1.6), with the item about discussing benefits scoring highest. In consultations where a decision aid was used (15, 42%), compared to the 21 usual care consultations, mean observer-assessed SDM scores (OPTION-12, ACEPP scores) were higher and antibiotic harms mentioned in all (compared to only 1) consultations. Patients generally reported high decision involvement and self-efficacy, and low decisional conflict.

**Conclusions:**

The extent of observer-assessed SDM between GPs and patients with ARIs was generally low. Balanced discussion of antibiotic benefits and harms occurred more often when decision aids were used.

**Electronic supplementary material:**

The online version of this article (10.1186/s12875-018-0854-y) contains supplementary material, which is available to authorized users.

## Background

One of the main causes of increased antibiotic resistance is high levels of antibiotic use, with approximately 80% of antibiotic use occurring in the community [1]. Within primary care, acute respiratory infections (ARIs) are one of the most common reasons for an antibiotic prescription, even though antibiotics provide only small benefit and can cause harms [2–5].

General practitioners’ (GP) antibiotic prescribing behaviours are influenced by many factors, including diagnostic uncertainty, perceived patient pressure for antibiotics, and the need to maintain a good relationship with patients [6–9]. Many patients believe that antibiotics resolve symptoms, are necessary, and have no harms [10]. These beliefs contribute to some patients expecting, and sometimes requesting, antibiotics [10–12].

Shared decision making (SDM) is a process that involves clinicians and patients jointly participating in making a health decision, after having discussed the options and the benefits and harms of each option, and considered the patient’s values, preferences and circumstances [13–15]. For most ARIs, the choice about whether to treat with antibiotics, or not, is nearly at equipoise, with the benefits closely balanced by the harms. This makes consultations for ARIs ideally suited for SDM. When deciding about antibiotic use for ARIs, most patients want more involvement in the decision-making process and more opportunity to weigh up the benefits and harms of the options [16, 17]. A recent systematic review found that interventions to facilitate SDM reduced antibiotic prescribing for ARIs in primary care, compared with usual care, from 47 to 29% (risk ratio of 0.61; 95% confidence interval 0.55 to 0.68) [18]. However, there has been little exploration of the prevalence and nature of SDM in GP consultations for ARIs, including whether and how any patient decision aids may be used to facilitate SDM.

In a sample of consultations (where some GPs had been provided with ARI decision aids), we aimed to: 1) analyse the extent and nature of SDM in consultations between GPs and patients with ARIs, including if and how antibiotic benefits and harms are discussed; 2) explore the use of patient decision aids in ARI consultations and the communication of antibiotic benefits and harms with and without decision aids; and 3) explore patients’ perspectives of the decision-making process.

## Methods

### Design

This was an observational study that ran in parallel to an ongoing cluster randomised trial of three decision aids (for acute otitis media [AOM], acute sore throat, and acute bronchitis) and a brief GP SDM training package [19] (Australian New Zealand Clinical Trials Registry (ANZCTR) number: ACTRN12616000644460).

### Participants and setting

For the trial, general practices were recruited from established GP research networks, primarily in southeast Queensland, Australia. Practices whose GPs had already consented for the trial or its pilot were invited to participate in, and provide written consent for, this additional study during 2017. Practices were not eligible if they had participated in any other study where the main intention was to reduce antibiotic prescribing for ARIs. Patients were eligible to participate if they met the following criteria: 1) adult or parent of a child consulting a GP with one of three ARIs (AOM, acute sore throat, acute bronchitis) for the first time for that illness episode; 2) able to understand and read English; and 3) provided written informed consent.

Some GPs (in practices that had been randomised to the trial’s intervention group or had piloted the intervention) had previously been provided with: 1) three decision aids (one each for AOM, acute sore throat, and acute bronchitis), in printed form (single A4 page, double-sided and laminated) and in PDF (Additional files 1, 2 and 3); and 2) a USB-drive containing a 15-min video-based SDM training package that explained what SDM is, its use in ARI consultations, and a consultation demonstrating use of one of the decision aids. These GPs were given the intervention package and encouraged to use the aids during consultations with patients with ARIs whenever they felt it was appropriate. No further instruction or encouragement to use the aids or SDM strategies occurred. The GPs in practices randomised to the control group did not receive the training package or decision aids and continued providing their usual care.

### Procedure

The exact procedure for recruiting patients varied according to each practice’s preference. On recruitment days, at some practices, one of us (MB) approached only patients who were waiting to see the GPs who were participating. In other practices, all waiting patients were approached and asked if they were waiting to see one of the participating GPs (GP names were listed). If so, we proceeded with recruitment. Patient eligibility was determined by asking the patients if they were suffering from one of the following symptoms (sore throat, cough, ear pain), and confirmed afterwards by the clinician. If the patient was diagnosed as having an illness other than an eligible ARI, the recording was deleted. After written informed consent was provided, an audio-recording device was handed to the GP who began recording just before the patient entered their consulting room. After patients left the room, they were given a short questionnaire (< 5 min) to complete. It contained basic demographic questions and items from tools to measure their perspectives of involvement in the decision-making process, decisional conflict, and confidence in decision-making (see section below on patients’ perspectives).

### Outcome measures

#### The extent of SDM (observer-assessed)

Each consultation recording was analysed, by listening to the audio-recordings, by two independent raters using two measures. One measure was the 12-item Observing Patient Involvement (OPTION-12) scale, which has good discriminative validity, concurrent validity, and interrater and intra-rater reliability [20, 21]. It contains 12 items scored on a five-point scale: (0) the behaviour was not observed; (1) a minimal attempt is made; (2) the behaviour is observed with a minimal skill level; (3) the behaviour is executed to a good standard; and (4) the behaviour is executed to a high standard. Total scores were re-scaled to 0–100. A second measure was 5 items (1 subscale) of the Assessing Communication about Evidence and Patient Preferences (ACEPP) tool. This was used as the OPTION scale does not specifically evaluate communication of the quantitative benefits and harms of the options. It has good reliability and has been used previously to assess evidence communication in consultations [22, 23]. The items rate clinicians’ performance in describing the benefits/harms in terms of patient outcomes, the likelihood of benefits/harms, and the evidence source. Items were scored as: the behaviour was not observed (0); behaviour was observed at a basic level (0.5); or observed to an extended level (1).

To establish scoring reliability, three of us (MB, EG, TH) independently rated an initial sample of recordings and responses were discussed until agreement was reached. Two of us (MB, EG) independently rated the remainder. Any rating discrepancies were resolved by a third person (TH). The two raters also extracted verbatim any mention of antibiotic benefits and harms.

#### Patients’ perspectives

Patients’ perceptions of their involvement in the decision-making process were measured using the CollaboRATE-5 scale (score range 0 to 5) [24, 25]. It asks three questions about what occurred in the consultation: 1) deliberation of the health issue, 2) exploration of patient preferences, and 3) integration of patient preferences [25]. The scale has demonstrated significant discriminative validity, excellent intra-rater reliability and concurrent validity with other measures of SDM [24].

Decisional conflict is a condition of uncertainty about options involving trade-offs and potential for regret. It was measured using the 10-item low literacy decisional conflict scale [26]. In this study, patients’ feelings conflict about whether they felt that their decision (using antibiotics or not) was the best for them was assessed. The scale has good validity and reliability [26]. The low-literacy version uses a question-and-answer format with three response options (yes, no, unsure), with scoring from 0 (low decisional conflict) to 100 (high decisional conflict) [27].

Patients’ confidence in decision-making was measured using four items from the decision self-efficacy scale [28], which has high internal consistency [29]. Scoring of each item is from 0 (not at all confident*)* to 100 (very confident*)*.

### Data analysis

We calculated descriptive statistics (mean, standard deviation, range) for each outcome measure. Data were analysed using IBM SPSS (version 23). Benefits and harms of antibiotics mentioned were categorised into similar groups, by description level as per ACEPP scoring, and by whether a decision aid was used.

We present the results for the whole sample in line with our original aims. However, to explore the impact of decision aids, we also present the data separately for those consultations in which a decision aid was used and not used, along with mean differences and 95% confidence intervals.

## Results

Ten general practices (3 intervention, 5 control), involving 44 GPs, that had already consented to participate in the main trial or piloting of the decision aids (2 practices) by the time that recruitment for this study commenced were invited to participate in this additional study. Of these, 5 practices and 19 GPs provided consent. During the recruitment period, 208 patients were approached and 41 met the inclusion criteria. Of these, 36 patients provided consent for the recording and 25 also agreed to complete the questionnaire. The main reason given for declining to complete the questionnaire was insufficient time. We recorded 36 consultations, involving 13 GPs - 20 were for acute bronchitis, 10 for acute sore throat, and 6 for AOM. Patient, GP, and consultation characteristics are presented in Table 1.

**Table 1:** Characteristics of the GPs, patients, and consultations

Characteristic	N^a^ (%)
GP gender – female	11 (61)
Patients	
Adults (Patient or parent)	18 (50)
Female	15 (83)
Age in years - median (min-max)	36 (18–77)
Children	18 (50)
Female	7 (39)
Age in years – median (min-max)	2 (0.8–15)
Condition	
Acute bronchitis	20
Acute sore throat	10
Acute otitis media	6
Decision aid used in the consultation	15 (42)
Consultation duration (minutes) - median (min-max)	9 (4–31)
Treatment decision (from analysis of consultation recording)	
Antibiotics	3
Delayed prescribing	7
No antibiotics	26
Treatment decision immediately post-consultation^b^ (as reported by patients)	
Antibiotics	5
No antibiotics	20

^a^This is the number of consultations, GPs, or patients

^b^Not all patients felt sufficiently decided to report their treatment decision during the post-consultation interview

### The extent of observer-assessed SDM

**Fig. 1:**
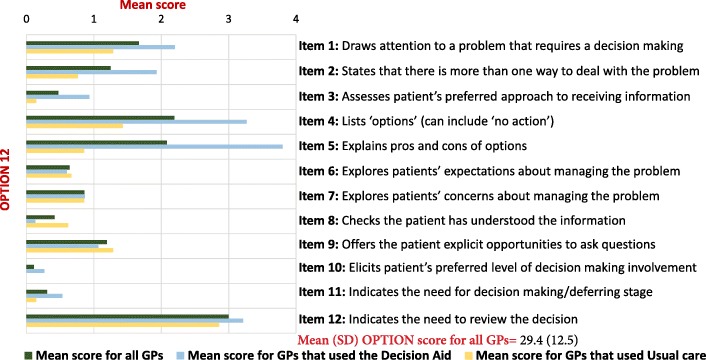
Mean scores of OPTION 12 items

**Fig. 2:**
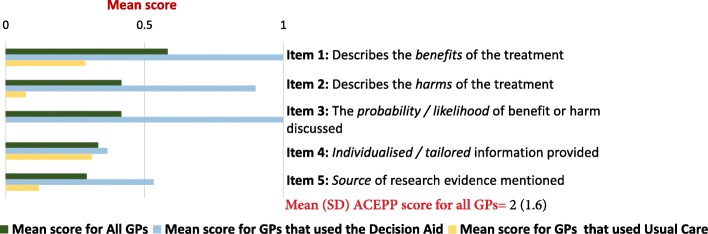
Mean scores of ACEPP items about communication of benefits and harms

The mean (SD) total OPTION score was 29.4 (12.5; range 4–54) (on a 100-point scale). The two highest scoring items were Item 12 (clinician indicates the need to review the decision) (mean = 3, SD = 1.5) and Item 4 (clinician lists ‘options’) (mean = 2.2, SD = 1.5). The two lowest scoring items were Item 10 (clinician elicits patient’s preferred level of involvement in decision making) (mean = 0.1, SD = 0.3), and Item 11 (clinician indicates the need for a decision making) (mean = 0.3, SD = 0.5) (Fig. 1). The mean (SD) total ACEPP score was 2 (1.6) (on a 5-point scale), with Item 1 (clinician describes the treatment benefits) scoring the highest (mean = 0.6, SD = 0.5) (Fig. 2).

In consultations in which a decision aid was used (*n* = 15), the mean (SD) total OPTION score was 38.8 (6.5), compared to 22.7 (11.5) for those (*n* = 21) in which an aid was not used - a mean difference of 16 (95% CI 9.4–22.7). Similarly, the mean (SD) ACEPP score in consultations where an aid was used was 3.8 (0.5) which was higher than those which did not 0.8 (0.8) - a mean difference of 3 (95% CI 2.6–3.5).

### Discussion of antibiotic benefits and harms

Table 2 contains verbatim examples of how antibiotic benefits and harms were presented within consultations, categorised by level of description. The three most commonly discussed harms were diarrhoea, rash, and antibiotic resistance. In the 21 consultations that did not use a decision aid, the potential harms were mentioned in only 1 consultation (with nausea mentioned). Conversely, in the 15 consultations in which a decision aid was used, at least one harm was mentioned in all of them. Two harms were mentioned in 14 (93%) and 3 harms in 13 (87%) of these consultations. When benefits were discussed, those mentioned were: that antibiotics help patients’ symptoms resolve faster; and reduce symptom severity, and the chance of complications. Benefits and their likelihood were explained in all 15 of the consultations where a decision aid was used. Where aids were not used, benefits were mentioned in 7 (33%) of the 21 consultations, but the likelihood of benefits described in only 1.

**Table 2:** Verbatim examples of how the benefits and harms of antibiotics for ARIs were presented by GPs within the consultations, grouped by level of description and whether a decision aid was used

	Benefits of antibiotics	Harms of antibiotics	
		Side effects	Resistance
With decision aids (*n* = 15)	Benefits mentioned in **15 (100%) of 15** consultations	Side-effects mentioned in **15 (100%) of 15** consultations	Resistance mentioned in **10 (67%) of 15** consultations
	Mentioned to an extended level* (15/15) *Examples:*	Mentioned to an **extended level (7/15)** *Examples:*	Mentioned to an **extended level (5/10)** *Examples:*
	**GP D-2-6** “All the evidence shows if we have somebody with middle ear infection like what we have got here now…if you don’t give any antibiotics the infection lasts about 3.5 days in total. If you give antibiotics it reduces that by 12 h. It can cut off about 12 h of the symptoms by giving antibiotics, so giving antibiotics is of limited benefit” … “so, if we look at 100 kids who don’t take antibiotics, 84 will be better in 3 days. If we give antibiotics there is an extra 5 kids who would be better.” **GP B-1-2** “Most sore throats get better somewhere between 2 and 7 days and that is actually whether or not you get antibiotics. Even if it is a bacterial infection you get better without antibiotics. So the treatment options are to take antibiotics or to not take antibiotics … This is a graph that shows you how long a sore throat would last on average. So if you take antibiotics, generally the sore throat would last about 2.6 days so just over 2 and a half days. If you do not take antibiotics on average it will last about 3.3 days, so that means it last about 16 h longer without the antibiotics.”	**GP A-2-1** “What we are looking at over here is what the potential complications maybe with antibiotics. So people who do not take antibiotics, 20 out of a 100 will have some other problems associated with the illness. Whether it be vomiting, diarrhoea or rash. Whereas if we give you antibiotics, you are more likely to have side effects or complications. So 7 more people out of a 100 …will have these potential side effects of these antibiotics. There are also other harms with antibiotics which can be having an allergic reaction, it can be the cost of buying them, remembering to take them…”	**GP A-5-2** “The other concern as well is antibiotic resistance, meaning you know the long term implications, all the good bacteria in his system being exposed to antibiotics as well they can develop some resistance, so …[if] he got meningitis in the near time and needs antibiotics, taking some will not work, because of previous resistance” **GP B-1-2** “one of the problems that a lot of the bacteria that we have had in the community for years is getting stronger and stronger and resisting the antibiotics that we have got. So we are finding this is why this shows here that only a few people finding any benefit from taking the antibiotics because there is more and more resistance in the community… but we are finding increasingly is that the more we use them for infections that your body could probably fight them by yourself, we are actually, unfortunately, breeding bacteria that become stronger and stronger… and unfortunately at this point of time we have bacteria that is resistant to everything we have got and there is nothing new on the horizon vastly different from what we have got”
		Mentioned to a basic level** (8/15) *Examples:*	Mentioned to a **basic level (5/10)** *Examples:*
		**GP D-2-4** “… the only problem is it increases the number of people who get rash, diarrhoea, another side effects because of the antibiotics…” **GP A-3-1** “… but then you look at the side effects and we have got all these people who do not take antibiotics obviously no side effects… and in the antibiotics you get more side effects basically. So that’s each one of these little dots is someone who gets the side effect”	**GP D-2-9** “but in the big picture we are building on antibiotic resistance and you know we are coming to time where these things might not work for infections you got them to do” **GP D-2-7** “… and then you worry about antibiotic resistance and stuff like that”
Without decision aids (*n* = 21)	Benefits mentioned in **7 (33%) of 21** consultations	Side-effects mentioned in **1 (5%) of 21** consultations	**Resistance was not mentioned in any consultations**
	Mentioned to an **extended level (5/7)** *Examples:*	No **extended level** mentions	
	**GP C-1-1** “The evidence is that middle ear infection gets better 12 h to 24 h earlier if you give antibiotics and the pain is better 12 to 24 h if you give antibiotics”		
	Mentioned to a **basic level (2/7)** *Examples:*	Mentioned to a **basic level (1/1)** *Examples:*	
	**GP F-1-5** “…in which case antibiotics won’t do anything to get you better quicker”	**GP F-2-1** “And antibiotics would just give him side effects and upset his tummy”	

*Extended level: The clinician explains the benefits or harms of antibiotic treatment in a manner that is clear, with elaboration on the likelihood of these occurring, **Basic level: The clinician lists at least some of the benefits or harms of antibiotic treatment

### Patients’ perspectives of the consultation and decision-making process

The mean (SD) CollaboRATE-5 score for all consultations was 3.8 (0.4), representing high perceived patient involvement in the decision-making process. The mean (SD) Decisional Conflict score was 3.2 (8), indicating a low level of decisional conflict. Participants had high confidence in the decision made, with a mean (SD) decision self-efficacy scale score of 95 (10). There were minimal differences between the scores of patients who had, and had not, been presented with a decision aid during the consultation (Table 3).

**Table 3:** Mean (SD) scores of observer-assessed SDM scores and patients’ perspective of the consultation and decision-making process

Observer-assessed SDM scores (*n* = 36 consultations)			
	Total Mean (SD) score		
	All GPs (*n* = 36)	Usual Care (*n* = 21)	Decision Aids (*n* = 15)
OPTION-12 (0–100)	29.4 (12.5)	22.7 (11.5)	38.8 (6.5)
ACEPP (0–5)	2 (1.6)	0.8 (0.8)	3.8 (0.5)
Patients’ perspective scores of the consultation and decision-making process (*n* = 25 patients)			
	Mean (SD)		
	All patients (*n* = 25)	Usual Care (*n* = 16)	Decision Aids (*n* = 9)
CollaboRATE-5 mean encounter score (0–5)	3.8 (0.4)	3.9 (0.3)	3.7 (0.5)
Decisional Conflict Scale (0–100)	3.2 (8)	3.1 (7)	3.3 (10)
Decisional Self-efficacy (0–100)	95 (9.9)	96.5 (6.8)	92.4 (13.9)

## Discussion

### Summary

Our analysis of consultations between GPs and patients with ARIs found the extent of observer-assessed SDM was generally low and the communication of antibiotic benefits and harms was also often suboptimal. In consultations in which patient decision aids were used, the discussion of antibiotic benefits and harms was more frequent and more comprehensive. When decision aids were not used, antibiotic harms were rarely mentioned, and antibiotic resistance was never mentioned.

### Strength and limitations

Strengths of our study include minimising any bias from clinicians choosing which consultations to record as patient consent occurred before the consultations; two independent raters scoring the consultations; and obtaining patients’ perspectives. Limitations include the design (not a true randomised trial, although it is nested within one), which might have exaggerated the effects of the decision aids; the small number of consultations and that they may be non-representative; and GPs’ self-selection to participate in this additional study, which may have recruited those more confident and competent in SDM. The presence of the audio recorder in the consultation and the researcher in the waiting room may have resulted in performance bias, such as the Hawthorne effect, and inadvertently acted as a prompt for GPs to attempt or improve SDM. Also, results are limited to one country and clinicians participating may not be representative of those in other settings.

### Comparison with existing literature

We know of no other studies that have objectively analysed the extent of SDM in GP-patient consultations for ARIs. Although a recent systematic review [18] of trials whose interventions had aimed to increase SDM in ARI consultations in primary care found that these interventions decreased antibiotic prescribing, none of the 10 included trials actually objectively measured whether SDM improved as a result of the intervention.

Similarly low OPTION scores to those in this study have been reported in previous studies in different settings, such as outpatient cancer patients consulting their physicians [30], patients with back pain consulting their GPs [31], and patients consulting nutritionists about dietary treatment [32]. In a systematic review of studies that had used OPTION-12 to analyse consultations, OPTION Item 12 was one of the most consistently observed behaviours [33], and Item 10 score was very low, similar to our study.

### Implications for practice and research

When decision aids were used the extent of SDM increased, including a large improvement in the frequency and quality of the conversation about antibiotic benefits and harms. Having the options with their pros and cons clearly listed in a decision aid may act as a reminder for GPs to discuss them with patients. The better discussion of antibiotic benefits and harms, including explaining the size and/or likelihood of them, is also likely due to the aids containing a very synthesized summary of the evidence about antibiotic benefits and harms. GPs may be unaware of the empirical benefits and harms data of antibiotics for ARIs. While no studies have examined GPs’ knowledge of antibiotic benefits and harms, a study of paediatricians found they overestimate antibiotic benefits for ARIs [34] and generally, clinicians tend to have poor knowledge of treatment benefits and harms, overestimate benefits and underestimate harms [35].

Better benefit-harm perception by patients is necessary for informed decision making, and randomised trials have shown this improves when decision aids are used [36]. In ARI consultations, improving patient benefit-harm perception is particularly important because the evidence shows near-equipoise in the benefits-harms balance, patients overestimate the benefits of antibiotics for ARIs [17, 37], and they rarely hear about the harms. Correcting these misperceptions may break the cycle of patient expectations of antibiotics as a driver of antibiotic prescribing.

Antibiotic resistance is different from the side-effects that might typically be discussed by clinicians because it is not obviously an immediate or personal consequence for the individual patient. Many members of the public have misunderstandings about what antibiotic resistance is [38] and believe that it does not affect them [39]. However, it is a global problem that can affect anyone, even if indirectly, and it needs confronting. Consultations in which antibiotics are being considered for common ARIs are an ideal time to discuss antibiotic resistance as part of the benefit-harm trade-off of using antibiotics because this is an area of very high consumption. We found many missed opportunities for discussions about this to occur. Even when resistance was mentioned, discussion was usually brief and often not clear. Clinicians’ misunderstandings of antibiotic resistance have been reported in a systematic review [40].

Patients perceived that they had high involvement in the decision, despite observer-assessed SDM scores which were quite low. Reasons for this are not clear. Perhaps patients have not experienced consultations in which SDM was performed to a high level and they have low expectations of what patient involvement actually is, or perhaps the brief tool used with patients did not capture enough elements or enough similar elements that the observer-used measures did, such as whether benefits and harms were discussed. Patients also reported low decisional conflict and high confidence in their decision. This may reflect that the decision about whether to use an antibiotic for a minor illness is perceived by patients as a relatively simple one-off decision with low-stake harms. A trial of a decision aid and intense GP training to increase SDM for ARIs also reported low decisional conflict in patients in both control and intervention groups, with no statistically significant between-group difference [41].

## Conclusions

This study highlights that in this convenience sample of patients with ARIs who were seeing a GP, some elements of SDM occurred during the consultation, but that there is need for improvements in the extent to which SDM occurs during such consultations, including how antibiotic benefits and harms are discussed. Patient decision aids may be part of the solution to improving this, but further research about their effect and how to support GPs to discuss antibiotic resistance with patients is needed.

## Additional files

Additional file 1: Acute bronchitis decision aid. A decision aid on antibiotic use for patients with acute bronchitis in primary care. (PDF 134 kb)

Additional file 2: Acute otitis media decision aid. A decision aid on antibiotic use for patients with acute otitis media in primary care. (PDF 126 kb)

Additional file 3: Sore throat decision aid. A decision aid on antibiotic use for patients with sore throat in primary care. (PDF 129 kb)

## Abbreviations

ACEPP: Assessing Communication about Evidence and Patient Preferences
AOM: Acute otitis media
ARI: Acute respiratory infections
GP: General practitioners
SDM: Shared decision making
