# Supplementary material for: Shared decision making and antibiotic benefit-harm conversations: an observational study of consultations between general practitioners and patients with acute respiratory infections
Source: BMC Fam Pract. 2018 Oct 6;19:165. doi: 10.1186/s12875-018-0854-y (PMC6173855; doi:10.1186/s12875-018-0854-y)
Supplement: Supplementary file 1 — Acute bronchitis decision aid. A decision aid on antibiotic use for patients with acute bronchitis in primary care. (PDF 134 kb) [file 12875_2018_854_MOESM1_ESM.pdf]

Acute bronchitis: should I take antibiotics?

- This decision aid is to help you decide whether to use antibiotics when **you or your child** has acute bronchitis (acute cough).
- This can help you to talk and make a **shared decision** with your doctor about what is best for you or your child.

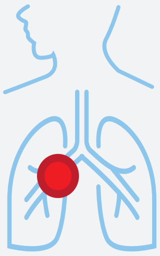

What causes acute bronchitis?

- It can be caused by a viral or bacterial infection. It is hard for your doctor to tell which it is.
- The infection is in the airway (bronchi) leading to the lungs. Acute means it is a short-term infection.

How long does the cough last?

- The cough will usually get better by about **10-20 days**, without needing to take antibiotics.

What are the treatment **options?**

There are 2 options that you can discuss with your doctor:

1. Not taking antibiotics

This means letting the cough get better by itself.

2. Taking antibiotics

Symptoms, such as fever, can be treated with over-the-counter medicines. They can be used with either option.

What are the likely **benefits and harms** of each option?

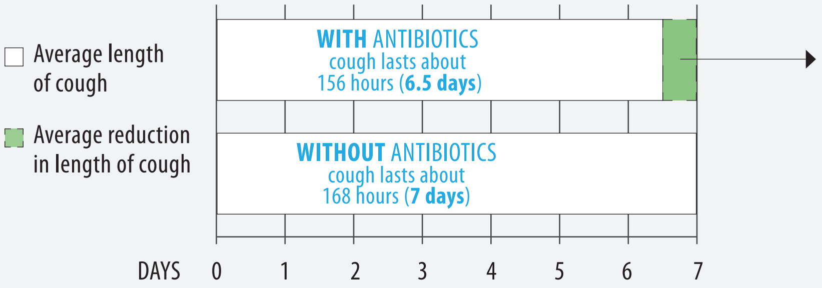

People who take antibiotics have the cough for only about **12 hours less** than people who do not.

These figures show what happens to people with acute cough who **do not** take antibiotics and those who **do**. Each circle is one person. We can't predict whether you will be one of the people who is helped or harmed.

- gets better by 1-2 weeks
- gets better by 1-2 weeks due to antibiotics
- not better by 1-2 weeks

- has problems
- has problems due to antibiotics
- no problems

100 people who **don't** take antibiotics — 100 people who **do** take antibiotics

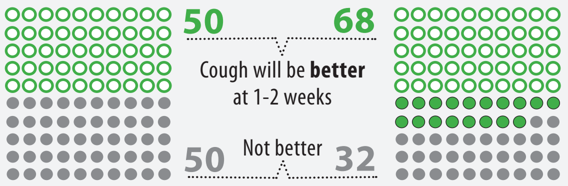

With antibiotics, **18 more people** will be better after 1-2 weeks.

100 people who **don't** take antibiotics — 100 people who **do** take antibiotics

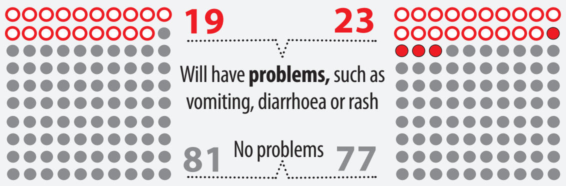

With antibiotics, **4 more people** will have problems like vomiting and diarrhoea. Other antibiotic harms are:

- the **cost** of buying them
- **remembering** to take them
- the risk of **antibiotic resistance** (see next page)

Where do these estimates of benefits and harms come from?

- They come from the most up-to-date medical evidence of benefits and harms about what works best. This is a review of 17 studies, and over 5000 people, that looked at antibiotic use in people with acute bronchitis.
- The quality of this research evidence is ranked as high. This means that further research is very unlikely to change these estimates.

Why might antibiotics be used?

If the infection is in the lung, it is called pneumonia. This is unlikely. However if it is pneumonia, it can be more serious. Your doctor may talk with you about why antibiotics might be needed. Coughing up coloured phlegm (spit) is not a sign that antibiotics are needed.

What is antibiotic resistance?

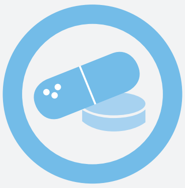

- Using antibiotics means the bacteria can develop resistance to the antibiotic.
- This means that **antibiotics will not work if you or your child needs them in the future** to treat a bacterial infection.
- A person who has recently used antibiotics is more likely to have resistant bacteria in their body.

Are there other things I can do?

- Fever is best treated with over-the-counter **paracetamol and/or ibuprofen**. Do not give more than the maximum recommended dose. Read the dose information on the packet.
- Aspirin should NOT be used with children who are younger than 16 years.
- Some people find that taking **honey** helps to settle the cough. Take 1-2 teaspoons, just before bedtime. The honey can be given in a drink such as warm water. Honey should not be given to children less than 12 months old.

When should you see a doctor and get further help?

If the person with the cough has any of these signs:

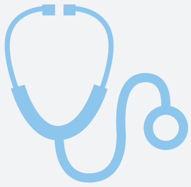

- Very drowsy
- Fast or difficulty breathing, wheezing, or shortness of breath
- Cold or discoloured hands and/or feet with a warm body
- Pain in the arms and/or legs
- Coughing blood
- Unusual skin colour (pale or blue) around the lips
- A rash that does not fade when the skin is pressed

Questions to consider when talking with your doctor

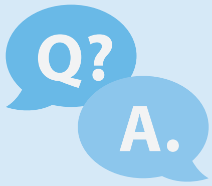

- ☐ Do I need antibiotics?
- ☐ What happens if I don't take antibiotics?
- ☐ Do I know enough about the benefits and harms of:
  - taking antibiotics?
  - not taking antibiotics?
- ☐ Am I clear about which benefits and harms matter most to me?
- ☐ Do I have enough information and support to decide?

References

1. Smith SM, Fahey T, Smucny J, Becker LA. Antibiotics for acute bronchitis. Cochrane Database of Systematic Reviews 2014, Issue 3. Art. No.: CD000245. DOI: 10.1002/14651858. CD000245.pub3. www.cochranelibrary.com

2. Gillies M, Ranakusuma A, Hoffmann T, Thorning S, McGuire T, Glasziou P, & Del Mar C. Common harms from amoxicillin: a systematic review and meta-analysis of randomized placebo-controlled trials for any indication. Canadian Medical Association Journal, 2015, 187; doi:10.1503/cmaj.140848.

3. Oduwole O, Meremikwu MM, Oyo-Ita A et al. Honey for acute cough in children. Cochrane Database of Systematic Reviews 2014, Issue 12:CD007094. doi: 10.1002/14651858. CD007094.pub4.

The information in this decision aid is provided for general information only. It is not intended as medical advice and should not be relied upon as a substitute for consultations with a qualified health professional who can determine you or your child's individual medical needs.

Last reviewed: November 2015. Update due: November 2017. Developed by Peter Coxeter, Professor Chris Del Mar and Professor Tammy Hoffmann - Centre for Research in Evidence-Based Practice, Bond University. Decision Aid development funded by the National Health and Medical Research Council (APP1044904).
