# Supplementary material for: Shared decision making and antibiotic benefit-harm conversations: an observational study of consultations between general practitioners and patients with acute respiratory infections
Source: BMC Fam Pract. 2018 Oct 6;19:165. doi: 10.1186/s12875-018-0854-y (PMC6173855; doi:10.1186/s12875-018-0854-y)
Supplement: Supplementary file 2 — Acute otitis media decision aid. A decision aid on antibiotic use for patients with acute otitis media in primary care. (PDF 126 kb) [file 12875_2018_854_MOESM2_ESM.pdf]

Middle ear infection: should my child take antibiotics?

- This decision aid is to help you decide whether to use antibiotics when **your child** has a middle ear infection.
- This can help you to talk and make a **shared decision** with your doctor about what is best for your child.

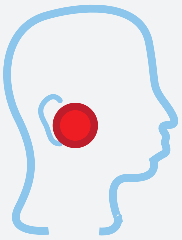

What causes middle ear infection?

- It can be caused by a viral or bacterial infection. It is hard for your doctor to tell which it is.
- It is also called 'acute otitis media'. Acute means it is a short-term infection.

How long does the earache last?

- Symptoms (such as earache) usually get better in 2 to 7 days, without antibiotics.

What are the treatment **options?**

There are 2 options that you can discuss with your doctor:

1. Not taking antibiotics
This means letting the infection get better by itself.

2. Taking antibiotics

Symptoms, such as pain and fever, can be treated with over-the-counter medicines. They can be used with either option.

What are the likely **benefits and harms** of each option?

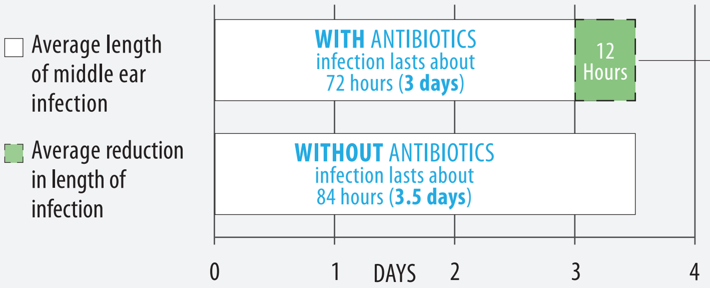

Children who take antibiotics have the earache for only about **12 hours less** than children who do not.

These figures show what happens to children with middle ear infection who **do not** take antibiotics and those who **do**. Each circle is one child. We can't predict whether your child will be one of the children who is helped or harmed.

- gets better by 2-3 days
- gets better by 2-3 days due to antibiotics
- not better by 2-3 days

- has problems
- has problems due to antibiotics
- no problems

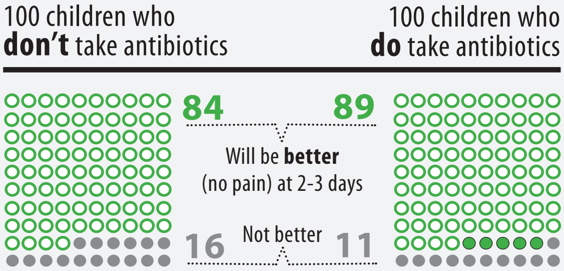

With antibiotics, **5 more children** will be better after 2-3 days.

After about **4 days** most children will be better anyway - without antibiotics.

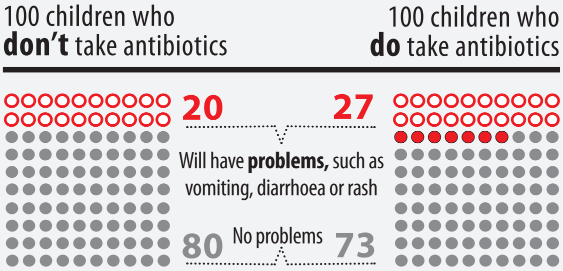

With antibiotics, **7 more children** will have problems like vomiting and diarrhoea. Other antibiotic harms are:

- the **cost** of buying them
- **remembering** to take them
- the risk of **antibiotic resistance** (see next page)

Where do these estimates of benefits and harms come from?

- They come from the most up-to-date medical evidence of benefits and harms about what works best. This is a review of 13 studies, and over 3,400 children, that looked at antibiotic use in children with middle ear infection.
- The quality of this research evidence is ranked as high. This means that further research is very unlikely to change these estimates.

Why might antibiotics be used?

There might be a special reason why your doctor may suggest antibiotics, such as in people who are more likely to get complications. This can be Indigenous children and children who are under 2 years of age.

What is antibiotic resistance?

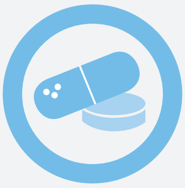

- Using antibiotics means the bacteria can develop resistance to the antibiotic.
- This means that **antibiotics will not work if your child needs them in the future** to treat a bacterial infection.
- A person who has recently used antibiotics is more likely to have resistant bacteria in their body.

Are there other things I can do?

- Pain and fever are best treated with over-the-counter **paracetamol and/or ibuprofen**. Do not give more than the maximum recommended dose. Read the dose information on the packet.
- Aspirin should NOT be used with children who are younger than 16 years.

When should you see a doctor and get further help?

If the child with the middle ear infection has any of these signs:

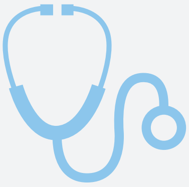

- Very drowsy
- Fast or difficulty breathing, wheezing, or shortness of breath
- Cold or discoloured hands and/or feet with a warm body
- A high fever (over 38.5°C)
- Pain in the arms and/or legs
- Unusual skin colour (pale or blue) around the lips
- A rash that does not fade when the skin is pressed
- Pain and tenderness of the bone behind the ear
- Blood or discharge from the ear

Questions to consider when talking with your doctor

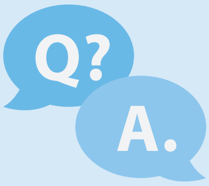

- ☐ Does my child need antibiotics?
- ☐ What happens if my child doesn't take antibiotics?
- ☐ Do I know enough about the benefits and harms of:
  - taking antibiotics?
  - not taking antibiotics?
- ☐ Am I clear about which benefits and harms matter most to me?
- ☐ Do I have enough information and support to decide?

References

Venekamp RP, Sanders S, Glasziou PP, Del Mar CB, Rovers MM. Antibiotics for acute otitis media in children. Cochrane Database Syst Rev 2015;1:CD000219. www.cochranelibrary.com

The information in this decision aid is provided for general information only. It is not intended as medical advice and should not be relied upon as a substitute for consultations with a qualified health professional who can determine you or your child's individual medical needs.

Last reviewed: November 2015. Update due: November 2017. Developed by Peter Coxeter, Professor Chris Del Mar and Professor Tammy Hoffmann - Centre for Research in Evidence-Based Practice, Bond University. Decision Aid development funded by the National Health and Medical Research Council (APP1044904)
