# Supplementary material for: Shared decision making and antibiotic benefit-harm conversations: an observational study of consultations between general practitioners and patients with acute respiratory infections
Source: BMC Fam Pract. 2018 Oct 6;19:165. doi: 10.1186/s12875-018-0854-y (PMC6173855; doi:10.1186/s12875-018-0854-y)
Supplement: Supplementary file 3 — Sore throat decision aid. A decision aid on antibiotic use for patients with sore throat in primary care. (PDF 129 kb) [file 12875_2018_854_MOESM3_ESM.pdf]

Sore throat: should I take antibiotics?

- This decision aid is to help you decide whether to use antibiotics when **you or your child** has a sore throat.
- This can help you to talk and make a **shared decision** with your doctor about what is best for you or your child.

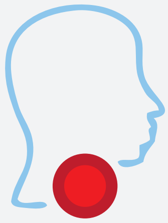

What causes sore throat?

It can be caused by a viral or bacterial infection. It is hard for your doctor to tell which it is.

How long does sore throat last?

- Symptoms will usually get better in 2 to 7 days, without taking antibiotics.

What are the treatment **options?**

There are 2 options that you can discuss with your doctor:

1. Not taking antibiotics

This means letting the infection get better by itself.

2. Taking antibiotics

Symptoms, such as pain and fever, can be treated with over-the-counter medicines. They can be used with either option.

What are the likely **benefits and harms** of each option?

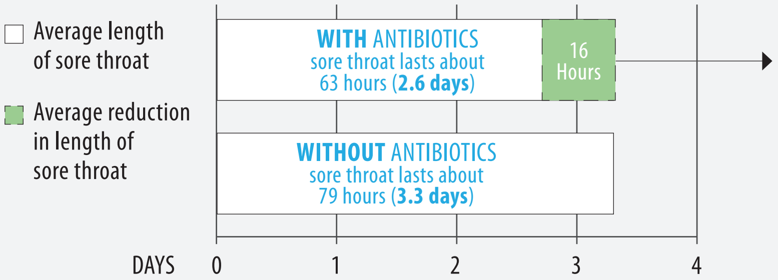

People who take antibiotics have the sore throat for only about **16 hours less** than people who do not.

These figures show what happens to people with sore throats who **do not** take antibiotics and those who **do**. Each circle is one person. We can't predict whether you will be one of the people who is helped or harmed.

- gets better by 3 days
- gets better by 3 days due to antibiotics
- not better by 3 days

- has problems
- has problems due to antibiotics
- no problems

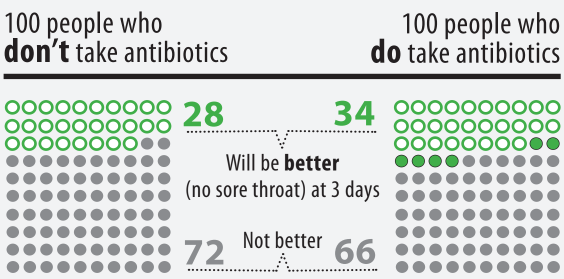

With antibiotics, **6 more people** will be better after 3 days.

Most people will be better after about **4-7 days** anyway - without taking antibiotics.

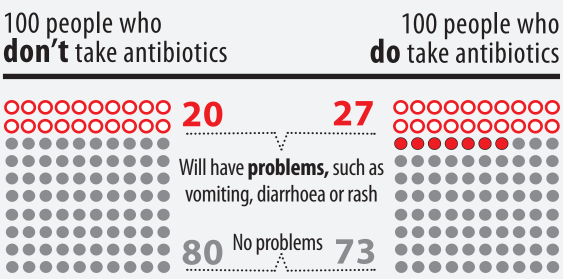

With antibiotics, **7 more people** will have problems like vomiting and diarrhoea. Other antibiotic harms are:

- the **cost** of buying them
- **remembering** to take them
- the risk of **antibiotic resistance** (see next page)

Where do these estimates of benefits and harms come from?

- They are from the most up-to-date medical evidence of benefits and harms about what works best. This is a review of 27 studies, and almost 13,000 people, that looked at antibiotic use in people with sore throat.
- The quality of this research evidence is ranked as high. This means that further research is very unlikely to change these estimates.

Why might antibiotics be used?

There are a few special reasons why your doctor might suggest antibiotics. This might be if the sore throat is caused by a dangerous, but rare, type of bacterium. Or in people who are at a high risk of complications, such as Indigenous people.

What is antibiotic resistance?

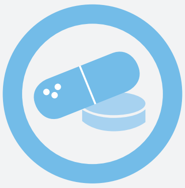

- Using antibiotics means the bacteria can develop resistance to the antibiotic.
- This means that **antibiotics will not work if you or your child needs them in the future** to treat a bacterial infection.
- A person who has recently used antibiotics is more likely to have resistant bacteria in their body.

Are there other things I can do?

- Pain and fever are best treated with over-the-counter **paracetamol and/or ibuprofen.** Do not give more than the maximum recommended dose. Read the dose information on the packet.
- Aspirin should NOT be used with children who are younger than 16 years.
- Gargle with warm salty water.
- Suck an ice cube or throat lozenge.

When should you see a doctor and get further help?

If the person with the sore throat has any of these signs:

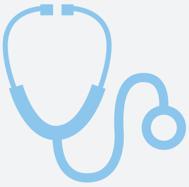

- Very drowsy
- Fast, noisy, or difficulty breathing, or shortness of breath
- Cold or discoloured hands and/or feet with a warm body
- Pain in the arms and/or legs
- Unusual skin colour (pale or blue) around the lips
- A rash that does not fade when the skin is pressed

Questions to consider when talking with your doctor

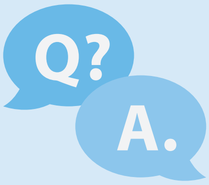

- [ ] Do I need antibiotics?
- [ ] What happens if I don't take antibiotics?
- [ ] Do I know enough about the benefits and harms of:
  - taking antibiotics?
  - not taking antibiotics?
- [ ] Am I clear about which benefits and harms matter most to me?
- [ ] Do I have enough information and support to decide?

References

1. Spinks A, Glasziou P, & Del Mar C. Antibiotics for sore throat. Cochrane Database of Systematic Reviews, 2013. 11: CD000023. www.cochranelibrary.com

2. Gillies M, Ranakusuma A, Hoffmann T, Thorning S, McGuire T, Glasziou P, & Del Mar C. Common harms from amoxicillin: a systematic review and meta-analysis of randomized placebo-controlled trials for any indication. Canadian Medical Association Journal, 2015, 187; doi:10.1503/cmaj.140848.

The information in this decision aid is provided for general information only. It is not intended as medical advice and should not be relied upon as a substitute for consultations with a qualified health professional who can determine you or your child's individual medical needs.

Last reviewed: November 2015. Update due: November 2017. Developed by Peter Coxeter, Professor Chris Del Mar and Professor Tammy Hoffmann - Centre for Research in Evidence-Based Practice, Bond University. Decision Aid development funded by the National Health and Medical Research Council (APP1044904).
